# Association of Antibody Responses to the Conserved *Plasmodium falciparum* Merozoite Surface Protein 5 with Protection against Clinical Malaria

**DOI:** 10.1371/journal.pone.0101737

**Published:** 2014-07-21

**Authors:** Ronald Perraut, Charlotte Joos, Cheikh Sokhna, Hannah E. J. Polson, Jean-François Trape, Adama Tall, Laurence Marrama, Odile Mercereau-Puijalon, Vincent Richard, Shirley Longacre

**Affiliations:** 1 Unité d'Immunologie, Institut Pasteur de Dakar, Dakar, Sénégal; 2 Laboratoire de Vaccinologie-Parasitaire, Institut Pasteur, Paris, France; 3 Laboratoire de Paludologie/ Zoologie Médicale, IRD, Dakar, Sénégal; 4 Unité d'Epidémiologie, Institut Pasteur de Dakar, Dakar, Sénégal; 5 Unité d'Immunologie Moléculaire des Parasites, CNRS URA 2581, Institut Pasteur, Paris, France; London School of Hygiene and Tropical Medicine, United Kingdom

## Abstract

**Background:**

*Plasmodium falciparum* merozoite surface protein 5 (PfMSP5) is an attractive blood stage vaccine candidate because it is both exposed to the immune system and well conserved. To evaluate its interest, we investigated the association of anti-PfMSP5 IgG levels, in the context of responses to two other conserved Ags PfMSP1p19 and R23, with protection from clinical episodes of malaria in cross-sectional prospective studies in two different transmission settings.

**Methods:**

Ndiop (mesoendemic) and Dielmo (holoendemic) are two Senegalese villages participating in an on-going long-term observational study of natural immunity to malaria. Blood samples were taken before the transmission season (Ndiop) or before peak transmission (Dielmo) and active clinical surveillance was carried out during the ensuing 5.5-month follow-up. IgG responses to recombinant PfMSP5, PfMSP1p19 and R23 were quantified by ELISA in samples from surveys carried out in Dielmo (186 subjects) and Ndiop (221 subjects) in 2002, and Ndiop in 2000 (204 subjects). In addition, 236 sera from the Dielmo and Ndiop-2002 surveys were analyzed for relationships between the magnitude of anti-PfMSP5 response and neutrophil antibody dependent respiratory burst (ADRB) activity.

**Results:**

Anti-PfMSP5 antibodies predominantly IgG1 were detected in 60–74% of villagers, with generally higher levels in older age groups. PfMSP5 IgG responses were relatively stable for Ndiop subjects sampled both in 2000 and 2002. ADRB activity correlated with age and anti-PfMSP5 IgG levels. Importantly, PfMSP5 antibody levels were significantly associated with reduced incidence of clinical malaria in all three cohorts. Inclusion of IgG to PfMSP1p19 in the poisson regression model did not substantially modify results.

**Conclusion:**

These results indicate that MSP5 is recognized by naturally acquired Ab. The large seroprevalence and association with protection against clinical malaria in two settings with differing transmission conditions and stability over time demonstrated in Ndiop argue for further evaluation of baculovirus PfMSP5 as a vaccine candidate.

## Introduction

*Plasmodium falciparum* malaria is one of the most important causes of morbidity and mortality worldwide, currently killing over 650,000 people annually, primarily African children under 5 years old. While scaled up control measures have decreased malaria morbidity and mortality in many areas of Africa [1], these efforts are threatened by parasite drug-resistance and anopheles vectors' insecticide resistance [2], [3]. In addition, natural immunity is waning as a result of reduced exposure to the parasite [4] leaving endemic populations at increased risk. Development of novel tools is needed to achieve the objective of control and elimination, amongst which efficient malaria vaccines.

The protective role of antibodies against blood stage malaria has been demonstrated using passive immunisation *via* transfer of antibodies from hyperimmune African adults to *P. falciparum* patients [5], [6]. However, it remains unclear which of the many antibody specificities present in hyperimmune sera are implicated in protection, information of great relevance for vaccine development. One approach to this problem is to investigate relationships between the antibody response to specific plasmodial antigens and the immune status of individuals naturally exposed to malaria in endemic areas.

Clinical symptoms of malaria occur during the blood stage of the parasitic cycle, during which asexual merozoites invade red blood cells, multiply intra-cellularly and egress to reinvade new cells in a cyclical process. Erythrocyte invasion is a rapid, multi-step process involving a number of merozoite membrane proteins accessible to immune effectors such as antibodies and complement [7]. Many merozoite surface proteins (MSPs) are anchored to the plasma membrane by a C-terminal glyco-lipid moiety (glycosyl-phosphatidyl-inositol, GPI), often attached to epidermal-growth factor (EGF)-like domains [7], [8].

The first identified and most studied MSP is merozoite surface protein 1 (MSP1), a 200 kDa protein proteolytically processed to a conserved C-terminal GPI anchored moiety of around 19 kDa called MSP1p19 composed of two adjacent EGF-domains [9], [10]. Naturally acquired antibodies binding *P. falciparum* MSP1p19 are major contributors to invasion inhibitory activity present in the serum of immune adults [11] and are correlated in an age-independent manner with clinical protection in endemic areas [12], [13], [14]. However, MSP1 is only one of several merozoite based immune targets [15], [16] and it is important to identify additional surface antigens of potential interest for development as vaccine candidates [17].

One such target of interest is *P. falciparum* MSP5. The *msp5* gene codes for a 272-residue protein with a C-terminal EGF-like domain and a GPI attachment motif [18]. While MSP5 function in *P. falciparum* is unknown, it is apparently not critical for parasite survival since viable knock-out mutants can be isolated with no apparent growth defect, at least under *in vitro* culture conditions [7]. However, MSP5 displays a surprising lack of population polymorphism in a parasite species renowned for its immune evasion strategy [8], [19], [20] and this feature is of particular interest for a vaccine designed to confer broad cross-strain protection. Nevertheless, there has been a notable paucity of epidemiological data monitoring antibody responses to PfMSP5 in the sera of endemically exposed populations, with only one recent publication on the subject [21].

The aim of the present study was to evaluate the relationship between *P. falciparum* MSP5 antibodies in sera from Senegalese endemic inhabitants using a baculovirus recombinant PfMSP5, and the protection status of the same individuals with regards to clinical malaria episodes and evaluate how this response vis a vis a response to other low polymorphic Ags already explored in this setting i.e. MSP1p19 and R23, an infected red blood cell associated repeat Ag [22]. The study was based on prospective serological studies carried out in two rural villages with similar socio-economical levels located 15 km apart in the same district of southern Senegal. Both villages participate in a longitudinal follow-up with the same protocol, but differ in transmission conditions, with holoendemic conditions in Dielmo and mesoendemic conditions in Ndiop. We measured the antibody response to MSP5 in sera collected in Dielmo and Ndiop before the transmission season, and all participants were monitored for clinical episodes by active daily case surveillance during the following 5.5 months [23]. We investigated whether the anti-MSP5 response was associated with protection against clinical malaria attacks during that period. Functionality of the antibodies was assessed using the *in vitro* assay based on neutrophil antibody-dependent respiratory bursts induced by opsonized merozoites (ADRB) [24] was investigated in a subset of samples. In complement, the stability over time of villagers' response to MSP5 and association with protection against malaria episode was studied through the analysis of 2000 data in Ndiop. The analysis was done in a multivariate model integrating MSP5 with other responses against MSP1p19 and R23.

The results provide a compelling case for the interest of baculovirus PfMSP5 as a blood stage malaria vaccine candidate.

## Methods

### Study area, study design and procedures

Subjects were recruited in Ndiop and Dielmo, two Senegalese malaria endemic villages participating in a long-term observational study on the acquisition and maintenance of natural immunity to malaria [25], [26]. The protocol was approved by the *ad hoc* Ethics Committee of the Ministry of Health. Each year, the project was reviewed by the Conseil de Perfectionnement de l'Institut Pasteur de Dakar and the assembled village population, and informed consent was individually renewed by all subjects. Individuals could withdraw from the study and the follow-up procedure at any time.

The protocol and objectives were carefully explained to the assembled villagers, and informed consent was obtained from all participants or their parents or guardians in the presence of an independent witness and confirmed by signature or by thumbprint on a voluntary consent form written in both French and Wolof, the local language.

Villagers were enrolled in the cross-sectional study in July 2002, before the rainy season *ie* before peak transmission in Dielmo, where transmission occurs year round due to a nearby stream, and before the transmission season in Ndiop (meso-endemic). These studies involved 186 and 221 healthy villagers, in Dielmo and Ndiop, respectively, i.e. 67% and 68% of the village population at that time. In Ndiop, Ab responses were also measured in 204 blood samples collected in July 2000, of which 141 were from villagers analysed in the 2002 survey. Characteristics of the three groups are summarized in Table 1, including the proportion of asymptomatic carriers with microscopically positive peripheral parasitemia at the time of blood sampling (range 0.5–80 trophozoites per 100 leucocytes). The detection threshold by experienced microscopists was 2 parasites per microliter i.e. 1 trophozoite per 200 fields of 0,5 µl of thick blood film from the systematic active follow-up protocol [23], [27]. After venous puncture, plasma and red blood cells were separated by centrifugation and stored at −20°C.

**Table 1:** Characteristics of populations analyzed in the study.

	Dielmo Year 2002	Ndiop Year 2002	Ndiop Year 2000
Number of villagers	186	221	204
sex ratio F/M	99/87	119/102	97/107
Mean age	28.6	24.2	24
Median age [range]	22.8 [3.4–80.5]	18.2 [3.4–76.9]	18.2 [3.6–75]
No Indiv. Hb AA/AS/AC	160/14/4	186/30/5	164/36/4
% individuals parasitemic*	45.9%	9.6%	18.1%
cumulative EIR**	295.5	17.9	50.7
No Indiv. included for clin. attacks	163	203	191
Overall No of clinical attacks	51	199	275

*Individuals with positive blood smear on the day of blood withdrawal.

**Cumulative Entomological Inoculation Rate measured during the 5 months follow-up.

Active clinical surveillance was carried out during the following 5.5-month transmission period. The protocol included notification of all febrile episodes and controlled use of anti-malarial drugs by the medical staff. Each villager was visited daily at home for clinical surveillance, and blood films were made in case of fever, and read extemporaneously, as described [13], [26], [28]. Because of different endemicity, parasite exposure and immune status, the definition of a malaria attack differs for Ndiop and Dielmo. In Ndiop, a clinical episode is defined as an association of symptoms suggesting malaria; fever (temperature ≥38°c), history of fever or diarrhea, with parasitemia >30 trophozoites/100 leukocytes whatever the age groups. In Dielmo, it is defined as an episode of fever (temperature >38.5 °C) associated with a parasite density exceeding an age-dependent pyrogenic threshold determined for this village. We used the threshold calculated after taking into account the impact of control measures implemented locally on malaria epidemiology [4], [29], [30]. Anti-malarial drugs were administered by the medical staff following each positive diagnosis of malaria [4], [23], [30].

The cumulative entomological inoculation rate (EIR) was monitored as described [25]. In Dielmo, malaria transmission was perennial with a cumulative EIR estimated at 295.5 infective bites per individual during the clinical follow up period of 2002 (July-Dec). In 2002, the cumulative estimated Ndiop EIR was 17.9 infective bites/individual from the beginning of September to the end of November, with no transmission in July, August and December. Two years before, the estimated EIR in Ndiop was substantially higher i.e. 50.75 infective bites/individual from end July to October, with no transmission recorded in November and December.

### Antigen and ELISA procedure

A synthetic gene coding for PfMSP5 (NF54 allele) was constructed with a codon usage adapted to baculovirus expression (manuscript in preparation). The construct encoded a polypeptide of 247 amino acids from Met-1 to Ile-247 with the C-terminal 25 amino acids mediating GPI modification deleted to allow protein secretion and replaced with a hexa-histidine tag to facilitate purification. Serine codons at positions S-80, S-99 and S-124 were replaced by alanine codons to prevent N-glycosylation. Recombinant baculovirus was produced using the pVL1393 transfer vector as previously described [31]. Recombinant PfMSP5 was secreted following infection of *Trichoplusia ni* insect cells (High Five, Invitrogen) with recombinant baculovirus, and purified by metalloaffinity chromatography using Talon, as previously described [32]. The baculovirus expression system has been shown to ensure optimal reproduction of conformational epitopes including EGF domains [10]. Recombinant PfMSP5 was diluted in PBS and used to coat Immulon-4 plates at a concentration of 0.5 µg mL^−1^.

IgG responses were quantified by ELISA in duplicate plasma samples diluted 1∶200 as previously described [13], [14], IgG subclasses were determined using human sub-class specific mouse mAbs and peroxidase-labeled goat anti-mouse IgG (1∶2000) from Sigma Chemicals (St Louis, Mo) after calibration for optimal concentrations [33], [34], [35]. A pool of 25 sera from clinically immune adults living in Dielmo and a pool of European and/or African non-immune sera were included as positive and negative controls, respectively, in each assay as standards for plate comparability. Results were expressed as OD ratio  =  OD sample / OD naive serum pool [13], [35]. Sera showing an OD ratio >2 corresponding to the signal of naive controls + 2 SD were considered sero-positive for prevalence calculations.

The Ags and ELISA test for IgG responses to PfMSP1p19 and to R23 was carried out as already described [13], [14], [22], [36], [37].

### Chemiluminescence monitoring of ADRB by neutrophils in the presence of merozoites

The ADRB assay has been detailed elsewhere [24]. Briefly, polymorphonuclear cells (PMNs) were harvested from pooled blood samples of 6–7 healthy donors by layering onto Ficoll-Hystopaque (density 1.077, Sigma) and collected after centrifugation 30 min at 400xg at the Ficoll-Hystopaque-RBC interface. After differential lysis of residual RBC using NH_4_Cl, 0.8 g.L^−1^ buffer, PMN were washed twice with Hank's balanced salt solution (HBSS), enumerated using trypan blue, and resuspended in PBS at 1–5.10^7^ cells mL^−1^.

Chemiluminescence was measured using opaque 96-well plates (Berthold), and a MicroLumat Plus 96 luminometer (Berthold). Merozoite pellets (40 µL) were incubated with 10 µL of test or control sera for at least 30 min at 37°C. Immediately following addition of PMN (100 µL) and isoluminol (100 µL of 1∶100 dilution in PBS of 4 mg.mL^−1^ stock in DMSO) plate reading started and continued for 1 h. To facilitate rapid handling, only 40–50 wells per plate were used, with the hyperimmune serum (HIS) internal control systematically deposited in the first and last wells.

Data are presented as a standardized activity index of ADRB calculated as: 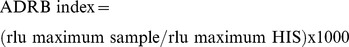
where rlu maximum HIS was the average of readout from the first and last wells on each plate. Only experiments in which the rlu maximum HIS was ≥100 (i.e. ≥6x background) were included in the analyses. An additional internal control with the same individual positive serum was included in each run.

### Statistical analysis

Antibody levels and growth and/or invasion inhibition in different groups were compared using the Wilcoxon signed rank test and the Spearman rank correlation test for non-normally distributed paired data. P values <0.05 were considered significant. A Poisson regression model was used to analyze the relationship between antibody response(s) and incidence of malaria attacks during the 5.5-month follow-up period. For the analysis, a *P. falciparum* malaria episode was defined as the presence of fever or symptoms suggesting malaria associated with >30 *P. falciparum* trophozoites/100 leukocytes, as determined by the same experienced microscopist. Malaria attacks were considered independent if separated by >15 days. For each villager, the follow-up time was calculated as the number of days actually spent in the village during the 5.5-month study and those absent more than 30 days during this period were excluded from the malaria incidence analysis. For individuals who received anti-malarial treatment following a malaria attack, a period of 15 days after the diagnosed attack was excluded from the days at risk calculation. The numbers of individuals and clinical episodes included in the calculations for each cohort are noted in Table 1. Age stratification was based on the age distribution of the parasitologic and clinical data available for Ndiop and Dielmo during the previous approximately 10 years of longitudinal follow up of both populations *ie* <15;15–30; >30 and <7;7–15; >15 years old age groups for Ndiop and Dielmo, respectively [14], [29], [30], [38], during which endemicity did not markedly change in each village [23], [28]. First-level interactions between variables were checked and included in the model when significant. The antibody level stratification was determined using Aikake's information criterion. Statistical analyses were performed with Egret 3.01 (Cytel), R and Statview 5.0 (SAS Institute) softwares.

## Results

### Seroprevalence and antibody levels to PfMSP5, PfMSP1p19 and R23

Table 2 shows that IgG reacting with baculovirus PfMSP5 was detected in 74% and 65% of villagers in Dielmo and Ndiop, respectively. Prevalence in Ndiop increased from 58% in the <15 y age-group to 70% in the >15 y group without reaching statistical significance (*P* = 0.058). In Dielmo, prevalence was high even in the youngest age group (<7 y). There was no significant difference in overall levels of anti-PfMSP5 IgG response (mean OD/median and OD ratios) between the two villages

**Table 2:** Prevalence and levels of antibody responses to PfMSP5 in the three cohorts.

	Ab responses against *Pf*MSP5		
	Dielmo Year 2002	Ndiop Year 2002	Ndiop Year 2000
Mean OD/median-[range]	0.41/0.32[0.01–1.7]	0.27/0.16 [0.01–1.46]	0.38/0.21 [0.01–2.63]
Mean ODratio/median-[range]	4.3/3.3 [1–17.1]	4.3/2.7 [1–22.8]	3.9/2.4 [1–23.2]
Prevalence of responders*	74%	65%	60%
Prevalence in younger individuals**	79% (n = 19)	58% (n = 85)	49% (n = 80)
Prevalence in older individuals	74% (n = 167)	70% (n = 136)	70% (n = 124)

*Individuals with positive IgG response =  ODratio>2 ie > OD of naive control + 2SD.

**Younger individuals : in Dielmo ie <7 years old; in Ndiop ie <15 years old.

**Figure 1:**
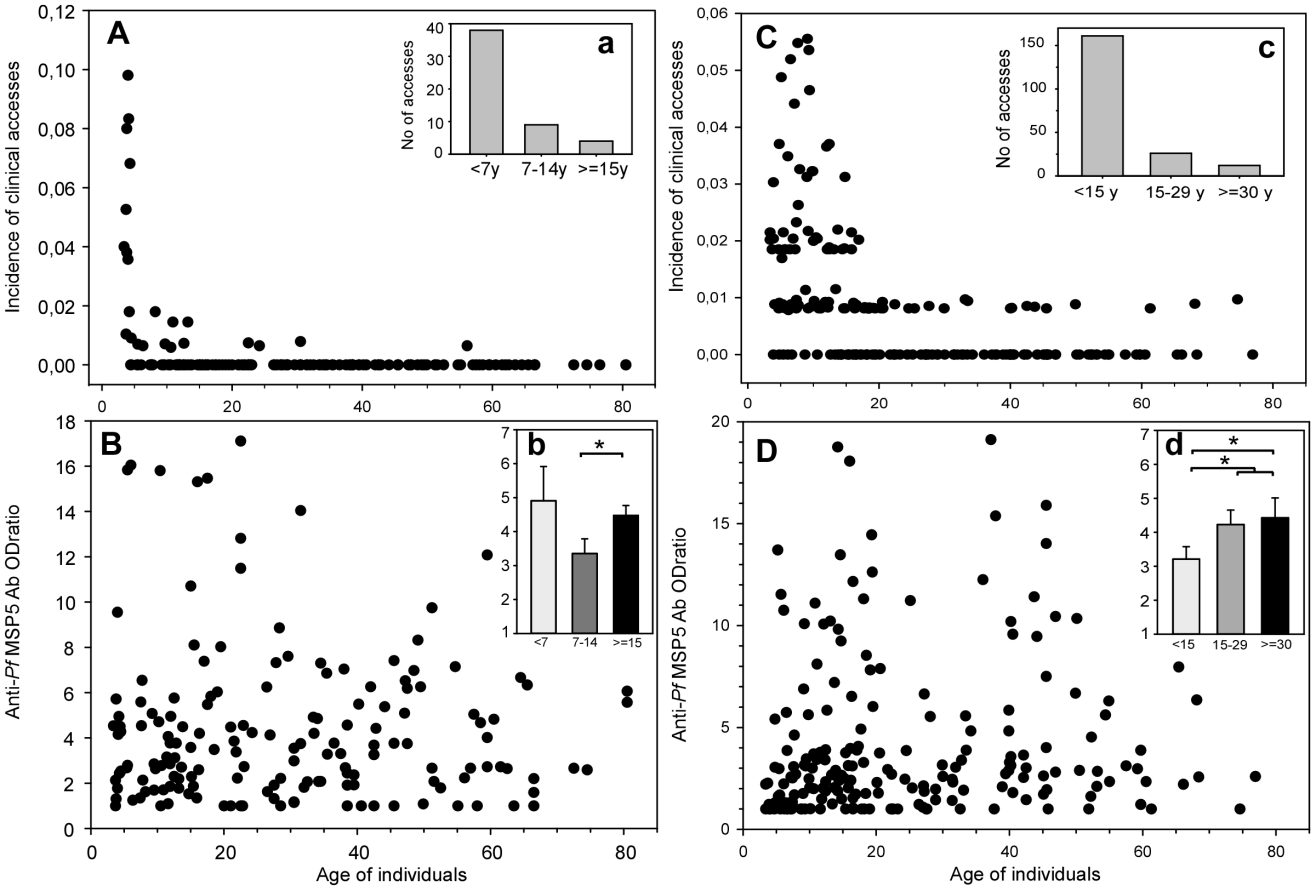
Age-related distribution of clinical episodes and IgG responses to PfMSP5. Distribution of clinical episodes and ELISA OD ratios reflecting IgG responses to PfMSP5 are plotted according to the age of individuals in Dielmo (**A** and **B**) and in Ndiop (**C** and **D**) in 2002. In captions are shown age-group distribution of number of clinical episodes in Dielmo (**a**) and Ndiop (**c**) and mean ODratio of anti-MSP5 IgG responses in Dielmo (**b**) and Ndiop (**d**). Groups were based on distribution of parasitologic and clinical data defined for both populations ie the <7, 7–14 and >15 y age-groups in Dielmo, and for the <15, 15–29 and >30 y age-groups in Ndiop. Brackets and asterisk indicate significant age-related differences (*P*<0.05) between levels of Ab responses.

The age distribution of clinical accesses and anti-PfMSP5 IgG responses in the two 2002 follow up studies is shown in Figure 1 (A and C). Inserts show the age-stratified clinical malaria incidence (panels a and c) and the anti-PfMSP5 IgG responses using the same clinical age-stratification (panels b and d). Significant differences were observed between the 7–14 y and >15 y groups in Dielmo, as well as between the <15 and ≥15–29 y groups in Ndiop. In Ndiop, there was a general trend towards higher anti-PfMSP5 responses in older age groups (*P*<0.05). In Dielmo, the higher responses observed in the <7 y group compared to the 7–14 y old children is possibly relate to substantial and recent exposure of individuals (134 positive smears [100% of individuals] and 47 clinical episodes [87% of individuals] in the 3 months before July), although this may reflect also a small sample size effect of the youngest age group (n = 19). A significant relationship between age and IgG response to PfMSP5 (*P*<0.01) was observed in Ndiop only, albeit with a low correlation coefficient (0.2–0.25).

Asymptomatic parasitemia on the day of sampling was associated with higher anti-PfMSP5 IgG only for the individuals from Ndiop (*P*<0.001). No correlations between antibody responses and gender or hemoglobin phenotype (*P*>0.05) were observed in either village.

In Table 3 are shown Levels and prevalence of IgG responses against PfMSP1p19 in the three settings, showing similar high levels of responses (mean ODratio above 7.9) and high prevalence in all age groups. There was a significant correlation between age of individuals and IgG to PfMSP1p19 in the 3 cohorts (*P*<10^−3^, correlation coefficient around 0.4). IgG response to R23, was substantially lower in the 2 villages in 2002 (mean OD/ODratio of 0.22/2.3 and 0.21/2.7) with a prevalence of 24% and 30% in Dielmo and Ndiop, respectively. Ab responses to R23 correlated with age in Ndiop only (*P*<10^−3^, correlation coefficient  = 0.27).

**Table 3:** Prevalence and levels of antibody responses to PfMSP1p19 in the three cohorts.

	Ab responses against *Pf*MSP1p19		
	Dielmo Year 2002	Ndiop Year 2002	Ndiop Year 2000
Mean OD/median-[range]	0.79/0.55[0.01–1.7]	0.86/0.80 [0–1.96]	0.94/0.79 [0.0–2.69]
Mean ODratio/median-[range]	7.9/5.3 [1–16.9]	6.0/5.5 [1–18.0]	7.9/6.7 [1–22.7]
Prevalence of responders*	73%	74%	78%
Prevalence in younger individuals**	68% (n = 19)	60% (n = 85)	65% (n = 80)
Prevalence in older inviduals	74% (n = 167)	83% (n = 136)	87% (n = 124)

*Individuals with positive IgG response =  ODratio>2 ie > OD of naive control + 2SD.

**Younger individuals : in Dielmo ie <7 years old; in Ndiop ie <15 years old.

### Comparison of anti-PfMSP5 antibodies in Ndiop

In Ndiop, it was possible to retrospectively evaluate the IgG responses to PfMSP5 of 204 villagers in a previous sampling conducted in July 2000, including 141 villagers who participated in the 2002 study. It was thus possible to compare the response in this sub-group of 141 villagers participating in both the 2000 and 2002 studies. Both seroprevalence (60% *vs* 71%) and antibody levels (ODratios  = 4.1 *vs* 4.4) showed very similar results in the 2000 and 2002 surveys with no significant difference by the Wilcoxon rank test.

**Figure 2:**
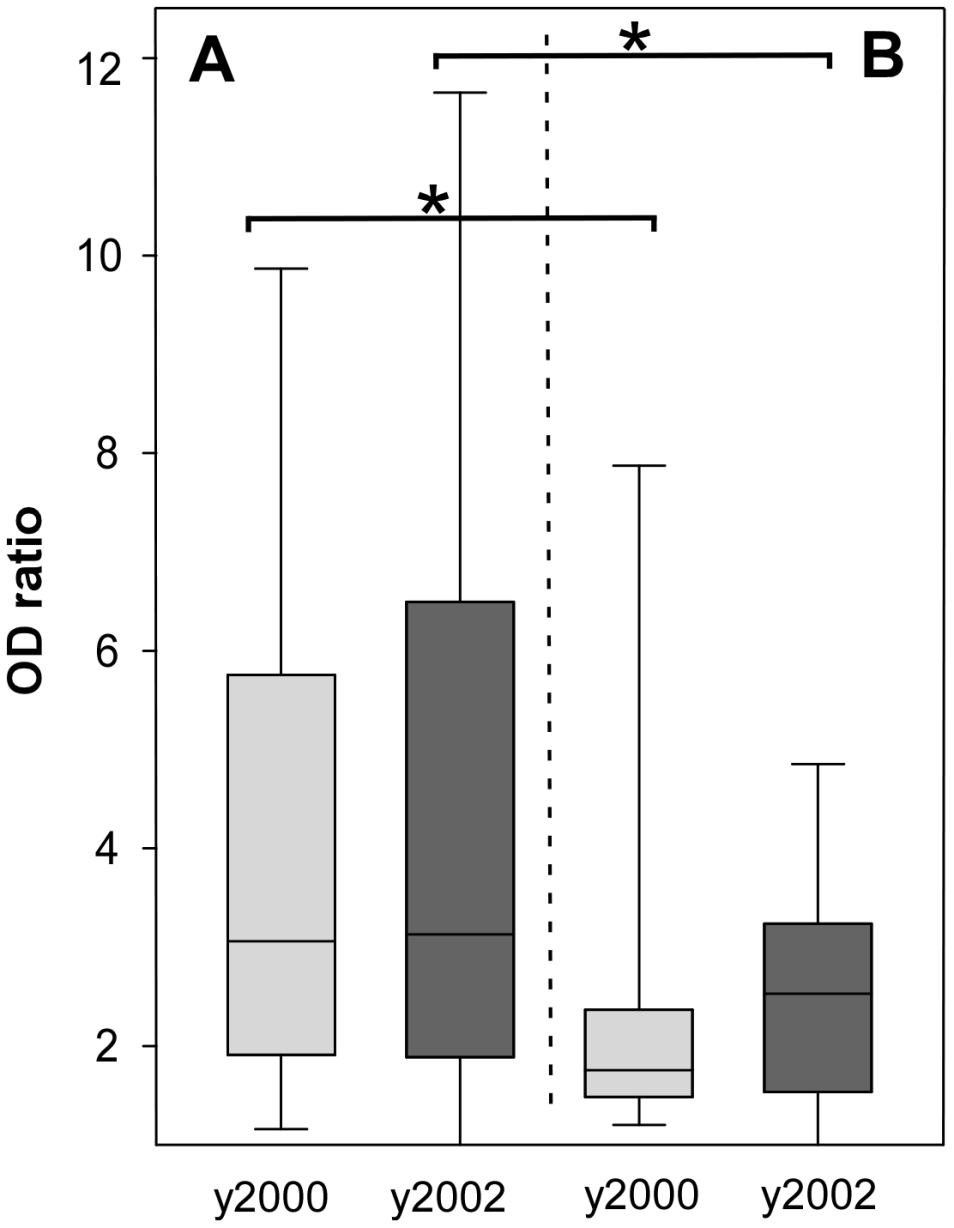
Longitudinal comparison of anti-PfMSP5 antibodies in Ndiop. Anti-PfMSP5 IgG responses for the 141 Ndiop villagers participating to two cross-sectional studies are shown. Ab responses were measured in both 2000 (light gray) and 2002 (dark grey). A box-whisker plot shows levels of Ab responses of individuals with the same profile *ie* positive or negative responses for both years (**A**). In part **B** are plotted Ab responses of 36 sera from individuals with discordant anti-PfMSP5 response in the two periods. Brackets indicate significant different levels between the two subgroups of villagers present in the two studies. The individuals with discordant responses from both surveys showed significantly lower Ab levels than those showing concordant responses (*P*<0.001).

Of the 141 individuals evaluated both years, 31 (22%) having a large age-distribution (5.2 to 50.3 years old in 2000) were negative in both surveys, whereas 74 others (52%) scored positive at both time points, showing comparable elevated levels of Ab with mean ODratios of 5.9 and 6.5 in 2000 and 2002, respectively In Figure 2 illustrating the degree of individual variation, panel A shows Ab responses for the 105 villagers with unchanged profiles in 2000 and 2002, and panel B shows responses for the 36 villagers scoring differently in the two studies, with 26 becoming positive between 2000 and 2002 while 10 were negative in 2002 after being positive in 2000.

### Isotype distribution of anti-PfMSP5 IgG

**Figure 3:**
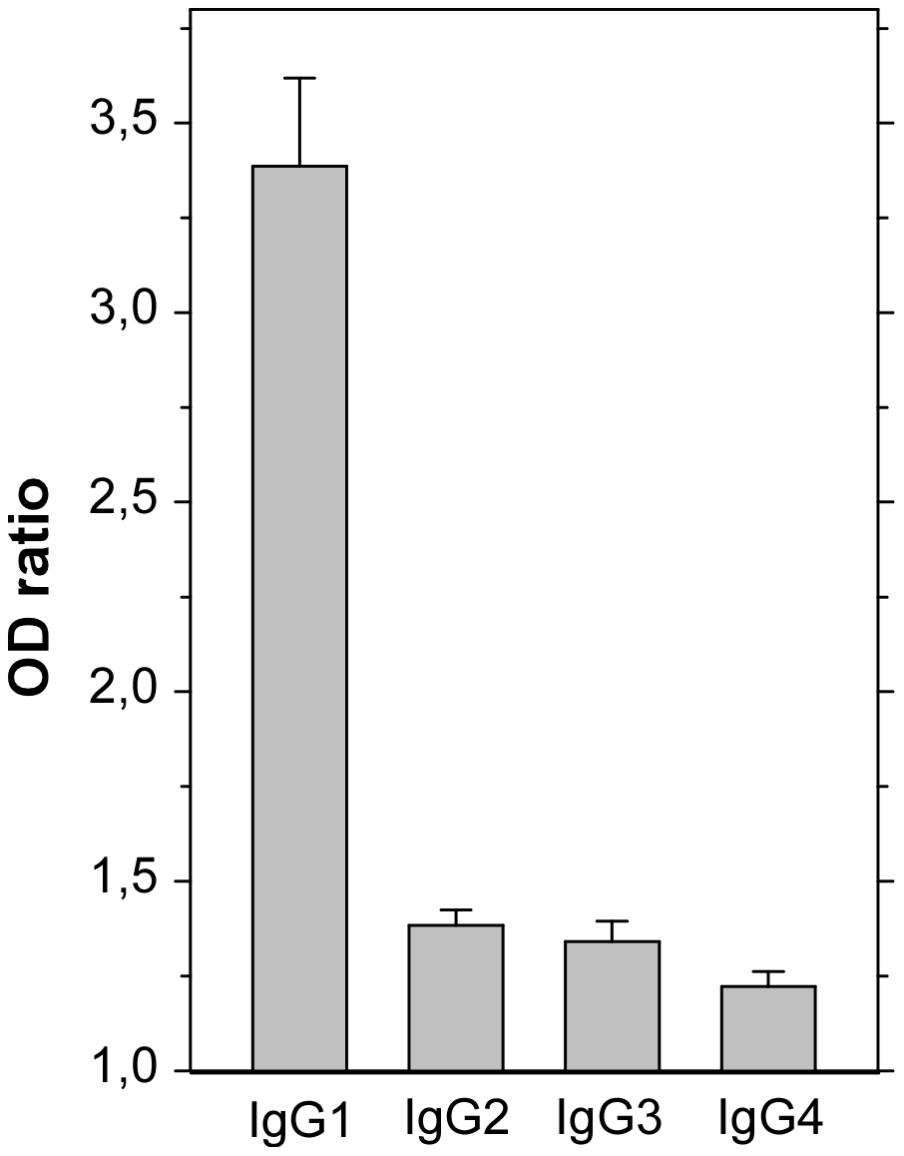
IgG isotype of anti-PfMSP5 responses. A set of 30 positive sera from all age groups were analyzed for anti-PfMSP5 IgG sub-classes as described in Methods. OD ratio results are plotted as bar charts + SD.

The isotype of IgG antibodies to PfMSP5 was investigated using a set of 30 positive sera with a similar age-distribution as the cohorts (mean age 24.5 y, median = 18.6 y, range 7.9–72.6 y). Anti-PfMSP5 antibodies were predominantly IgG1 with only low levels of other subclasses (Figure 3). IgG1 levels were unrelated to age, gender, hemoglobin type or microscopically positive, asymptomatic parasitemia at the time of blood sampling (P>0.05).

### Association of ADRB activity with age and anti-PfMSP5 antibodies

The antibody-dependent respiratory burst (ADRB) assay measures the capacity of antibodies to opsonize merozoites and induce a neutrophil respiratory burst, which is quantified by chemiluminescence. Because of its dependence on relatively intact merozoites [24], the assay is primarily a measure of functional activity of antibodies reacting with merozoite surface antigens. It was thus of interest to investigate the relationship between anti-PfMSP5 antibody levels and ADRB activity. Samples from cross sectional studies were randomly selected but matched with the age-group distribution of individuals from the 2002 studies. Altogether, 236 sera were tested for ADRB activity, including 120 from Dielmo (mean age 25.2; 3.4–80.5 y) and 116 from Ndiop (mean age 22.9; 3.9–76.9 y). In addition, 93 sera from the Ndiop 2000 sampling (mean age 22.9; 3.9–76.9 y) were assayed for ABRB activity. ADRB readout was expressed as a standardized value relative to a control pool of hyperimmune sera (ADRB index) [24]. Geometric mean values for the ADRB index were 255 (range 40–958), 201 (range 51–1721) and 504 (range 82–1809) respectively for the three set of samples from Dielmo-2002, Ndiop-2002 and Ndiop-2000, respectively.

In the three surveys, age and ADRB responses were significantly correlated: *P*<0.001 rho = 0.53; 0.41 and 0.44 for Dielmo-2002, Ndiop-2002 and Ndiop-2000, respectively, and ADRB indexes were significantly lower in the youngest age group (*P*<0.05).

**Figure 4:**
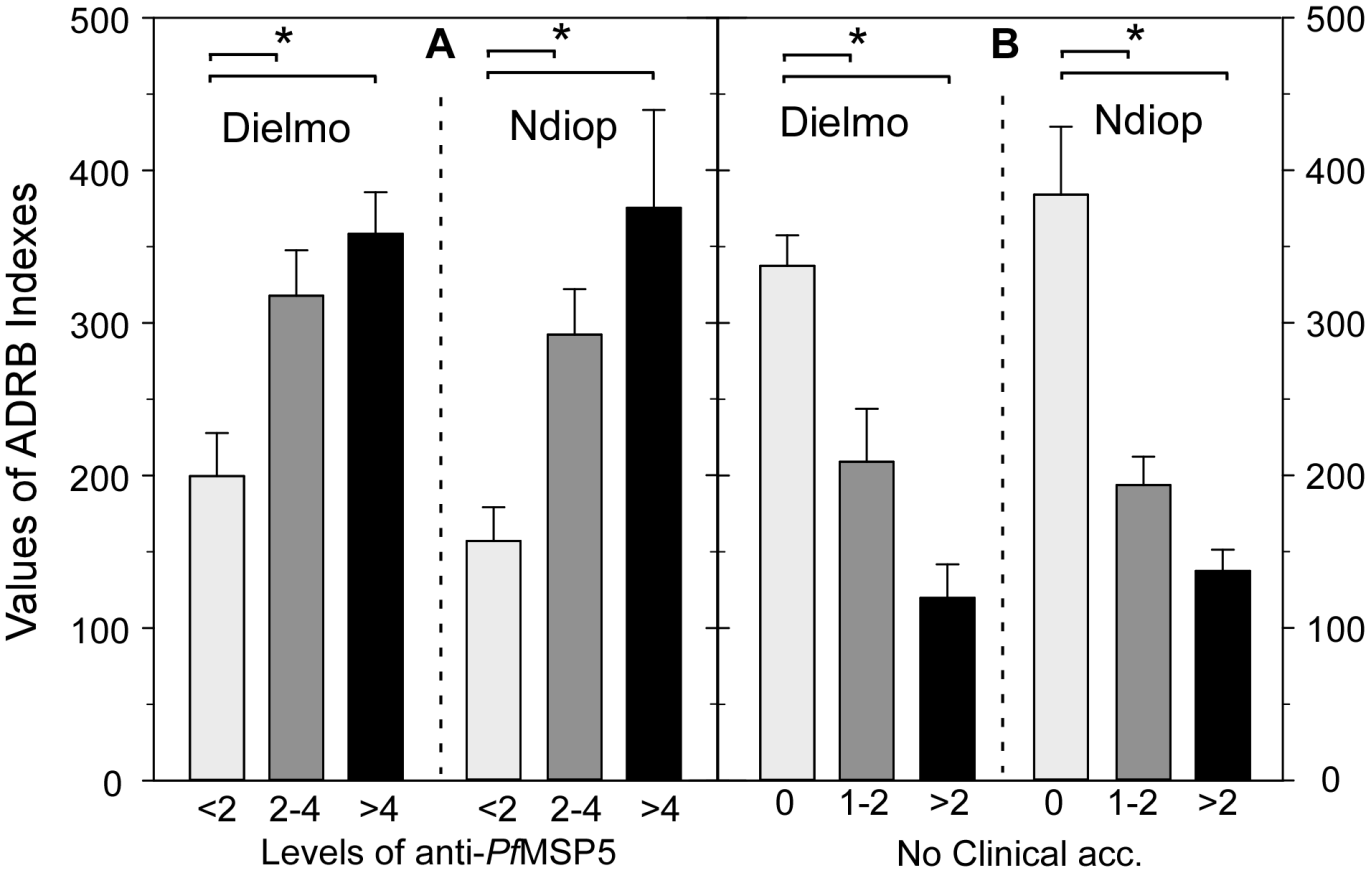
Relationship between ADRB functional antibody responses and anti-PfMSP5 IgG responses or malaria episodes. ADRB indexes were determined for 236 sera (120 for Dielmo-2002, 116 for Ndiop-2002) as described in Methods and compared (**A**) between individuals stratified by anti-PfMSP5 responses and (**B**) between individuals stratified by clinical outcome. In **A**, anti-PfMSP5 responses expressed as OD ratios <2 for negative (light grey bars), 2–4 for moderate (dark grey bars) and >4 for strong (black bars). In **B**, mean ADRB values are plotted by stratified clinical outcome: no (light grey), one or two (dark grey) and more than 2 clinical malaria episodes (black) during the 5.5 month follow up. Bracket and asterisks indicate significant differences (*P*<0.05).

ADRB indexes and PfMSP5 antibody responses were positively correlated (*P*<0.001 rho  = 0.33, 0.40 and 0.29 for Dielmo-2002, Ndiop-2002 and Ndiop-2000, respectively). When anti-PfMSP5 IgG OD ratios were stratified into negative (ODratio <2), median (ODratio 2–4) and high values (ODratio>4), they were positively associated with significantly different ADRB indexes in all cases (*P* = 0.01), suggesting a potential contribution of anti-PfMSP5 antibodies to this functional parameter (Figure 4A).

### Association of PfMSP5 antibodies with reduced incidence of clinical episodes

ADRB indexes were related to protection against clinical malaria during the 5.5 month follow-up. When stratifying into three clinical group, (0 *vs* 1–2 *vs* >2 cumulative clinical episodes), there was an inverse relationship with ADRB indexes (Figure 4B). ADRB indexes decreased as the number of clinical episodes increased in Dielmo-2002 and Ndiop-2002 (*P*<0.01) as well as in Ndiop-2000 (data not shown). This association was independent of age by the chi square test of independence (*P*<0.01).

The relationship between the anti-PfMSP5 response and incidence of clinical malaria during the 5.5-month follow-up period was analyzed using an age-adjusted Poisson regression model for each survey. Ab responses to R23 did not correlate with protection and were not included in the final multivariate model. In Dielmo-2002, clinical episodes were significantly associated with age (7–14 *vs* ≥15 y, RR^1^ =  8.43, *P*<0.001; 2–6 *vs* ≥15 y, RR^2^ = 87.54, *P*<0.001), but not hemoglobin phenotype or positive parasitemia at sampling. Dichotomization of the anti-PfMSP5 response based on negative *vs* positive sera was the best model for this cohort, showing a significant association with protection in the age-adjusted analysis (RR = 0.53 [CI 95% = 0.30–0.95]; *P* = 0.03).

In an age-adjusted model including both anti-PfMSP1 response (RR = 0.98, 95%CI [0.88-1.08]) and anti-PfMSP5 response (RR = 0.42, 95%CI [0.23–0.77]), only the anti-PfMSP5 responses were correlated with clinical malaria (P<0.01).

For the Ndiop-2002 cohort, variables correlated with clinical malaria were: (i) age (15–29 vs ≥30 y, RR^1^ = 2.75, *P* = 0.004; 2–14 vs ≥30 y, RR^2^ = 13.10; *P*<0.001); (ii) hemoglobin phenotype (AA vs AS, RR = 0.44; *P* = 0.004); (iii) positive parasitemia at sampling (RR = 1.56, *P* = 0.012) and (iv) the continuous anti-*Pf*MSP5 response (OD ratios) in an age-adjusted model (RR = 0.95 per unit lost; *P* = 0.002). In an age-adjusted model including both anti-PfMSP1 response (RR = 0.96, 95%CI [0.93–0.99]) and anti-PfMSP5 response (RR = 0.97, 95%CI [0.93–1.01]), only the anti-PfMSP1 response was correlated with clinical malaria (P-value = 0.04). Analysis of the Ndiop-2000 cohort showed similar results in age-adjusted model not including anti-PfMSP1 response. However, according with the age-adjusted model including both anti-PfMSP1 (RR = 0.97, 95%CI [0.95–0.99]) response and anti-PfMSP5 response (RR = 0.91, 95%CI [0.87–0.96]), showed that both anti-PfMSP1 and anti-PfMSP5 responses were correlated with clinical malaria (P-value = 0.02 and *P*<0.001, respectively).

**Figure 5:**
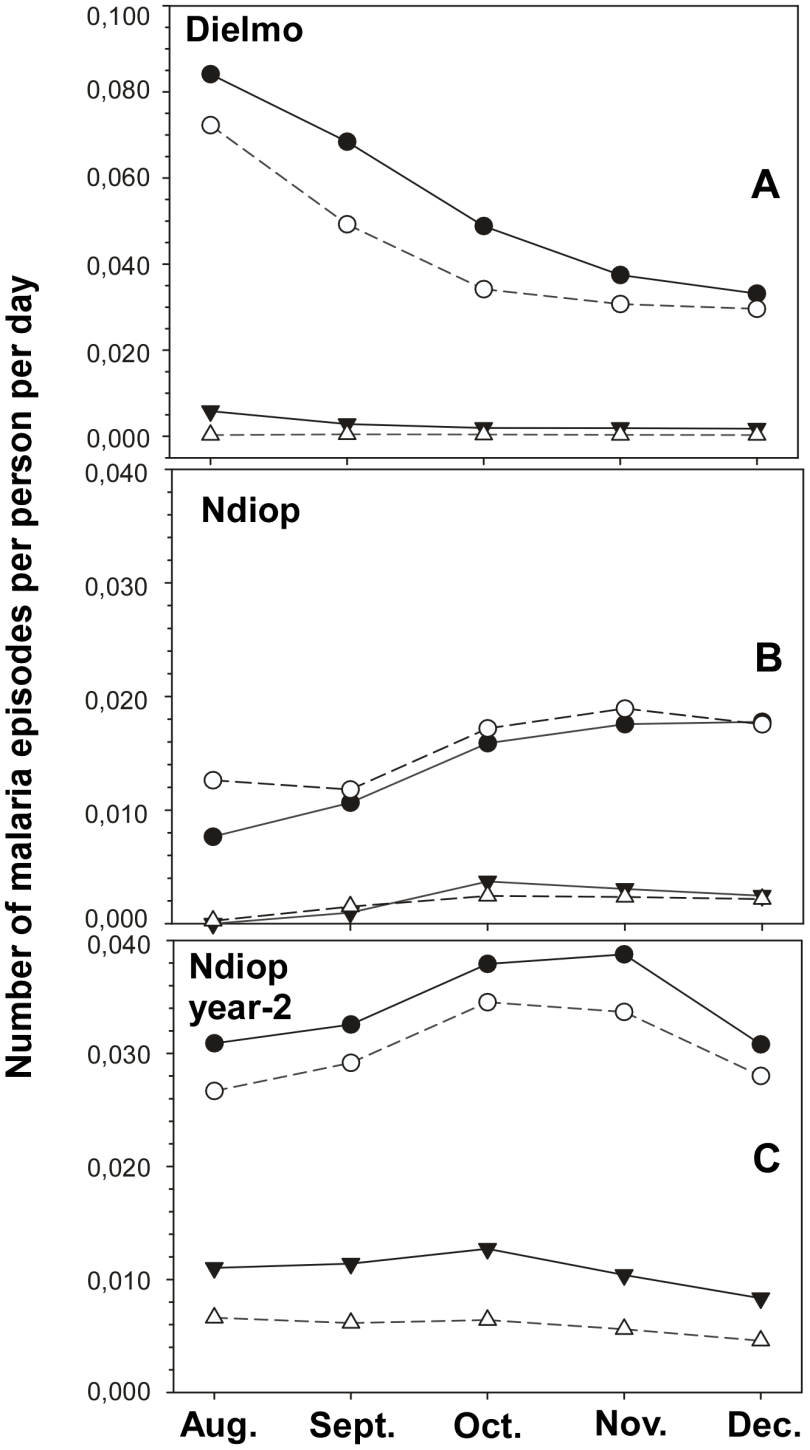
Association between anti-PfMSP5 IgG responses and cumulative incidence of clinical malaria episodes in different age groups. The cumulative monthly incidence of clinical attacks across the 5.5-month follow up was determined for individuals with dichotomized negative i.e. OD ratio ≤2 (filled symbols, solid lines) vs positive responders, i.e. OD ratio >2 (open symbols, dashed lines) against anti-PfMSP5 IgG for each cohort. Young individuals (circles) vs older (triangles) are shown for Dielmo-2002 (A: <7yo vs ≥7yo; EIR = 295.5), for Ndiop-2002 (B: <15yo vs ≥15yo; EIR = 17.9) and for Ndiop-2000 (C; EIR = 50.7).

Figure 5 shows the temporal incidence of number of malaria attacks over the 5.5-month follow-up period. Very consistent results have been obtained in the two settings and the two study periods.

Indeed, in both villages and age groups, a high OD ratio was associated with fewer clinical episodes through the 5.5 month follow-up period. These results are particularly marked in the age groups and study periods with higher malaria incidence, i.e. in the youngest most susceptible age group of both villages and in Ndiop 2000 (year-2) where transmission level was higher than in 2002. The only exception is observed in the oldest age group in Ndiop 2000 where the very low incidence can explain the difference with the other results (only 2 clinical malaria episodes per 1000 person-days for the ≥30 y age group).

## Discussion

The present study aimed to investigate the value of PfMSP5 as a blood stage malaria vaccine candidate by analyzing possible associations between naturally acquired antibodies to this antigen in endemic populations and susceptibility to clinical episodes of *P. falciparum* malaria. PfMSP5 has been relatively neglected in the literature compared to other *P. falciparum* MSP vaccine candidates such as PfMSP1, PfMSP2 and MSP3 or AMA-1 all of which are polymorphic and moreover induce allele-specific responses [39], [40], [41]. PfMSP5 appears to be relatively unique among blood stage surface antigens in exhibiting virtually no polymorphism. One hypothesis accounting for this remarkable conservation would be that PfMSP5 remains somehow undetected and as such unselected by naturally acquired antibodies. Clearly with 60–74% responders in this study, PfMSP5 does not escape the attention of the immune system. Prevalence was high and antibody levels were substantial. This indicates that other factors –possibly strong constraints on function- must account for the strong conservation of PfMSP5.

Two features of the approach used here were particularly important for maximizing the *in vivo* relevance of the results obtained. First, the recombinant PfMSP5 antigen used in the study was produced in the baculovirus expression system to optimize its resemblance to the native homologue, especially regarding conformational epitopes in the EGF domain and elsewhere in the molecule. Previous work with recombinant MSP1p19 has shown that this system appears to be particularly competent in achieving this objective [9], [10]. Nevertheless to prevent undesirable N-glycosylation the serine codons at three potential N-glycosylation sites were changed to alanine. A previous study on PfMSP5 antibody recognition used an *E. coli* recombinant antigen [21], which may not optimally reproduce some native conformational epitopes. This could account for some differences in results from the two studies relating to prevalence (39%–50% *vs* 60–74% here) and IgG isotype (IgG1 and/or IgG3 *vs* exclusively IgG1 here).

Secondly, the study was carried out in two endemic Senegalese villages with well-defined seasonal (Ndiop) and year-round (Dielmo) transmission, which have participated in a long-term cross-sectional prospective study with intensive follow-up for over 15 years. The study thus could rely on access to antisera from three different large cohorts of endemic donors and the protocol involved active case detection over a long 5.5-month follow-up with daily medical visits to each participant and the use of efficient diagnostic criteria to distinguish fevers due to malaria from other potential causes. These various factors provided the statistical power underlying many of the findings presented here.

IgG responses to PfMSP5 generally increased with age, although the youngest Dielmo age group had substantially high magnitudes, possibly due to the low number of group members and/or transient elevated responses by some susceptible individuals having experienced recent infection at this holoendemic site with frequent infective bites (a mean 295.5 infective bites for Dielmo-2002 compared to 17.9 and 50.75 for Ndiop in 2002 and 2000, respectively). The general trend associating older individuals with augmented antibody responses is a frequent observation with many plasmodial antigens in endemic areas, which likely reflects the probability of increased cumulative parasite exposure with age. It has been observed in this study in a very similar manner for IgG responses to PfMSP1p19 in all cohorts, and to a lesser extent for R23 in Ndiop only.

Anti-PfMSP5 responses were surprisingly stable in prevalence and magnitude over two years for the group of 141 individuals who were members of both the 2000 and 2002 Ndiop studies, the main difference being the 26% who became sero-positive with low OD levels during this interval. Since sampling occurred prior to the transmission season before any boosting by exposure to parasites, it is likely that the observed anti-PfMSP5 antibody levels persist for at least 6–7 months between transmission seasons, and may not change appreciably in the overall population from one season to the next. Thus, anti-PfMSP5 antibodies do not appear to be short-lived as suggested elsewhere [21], although the latter observation may pertain particularly to transient increases due to active infection.

When a cohort of 30 positive anti-PfMSP5 responder sera was analyzed for IgG isotype levels, IgG1 was by far the predominant species with only small amounts of IgG2, IgG3 or IgG4. The previous study measuring anti-PfMSP5 IgG isotype responses seemed to implicate also IgG3 [21], but it is difficult to compare results from the two studies because of differences in recombinant antigen used to measure responses, cohorts, and readout (prevalence versus levels). The IgG1 and IgG3 “cytophilic” isotypes couple adaptive and innate immune functions via Fcgamma receptors (FcgR) expressed on cellular effectors. In particular, merozoites opsonized with specific IgG1 and/or IgG3 are targets for antibody-dependent phagocytosis by neutrophils, which are highly professional phagocytes [42], [43]. IgG1 and/or IgG3 opsonized merozoites are also able to elicit respiratory bursts by neutrophils, an activity that has been correlated with protection from clinical episodes of *P. falciparum* malaria [24]. Indeed, we have shown here a significant positive association between stratified anti-PfMSP5 antibody levels and antibody-dependent respiratory burst (ADRB) activity by neutrophils *in vitro*, suggesting that PfMSP5 antibodies might be a component of this mechanism. In this study, a similar relationship between ADRB and IgG to baculovirus derived PfMSPp19 Ag was evidenced. We observed in 2002 a significant relationship of ADRB with clinical protection in an age-adjusted manner in the two villages [24], and approximately 30% of ADRB activity has been found related to PfMSPp19 Ag after selective depletion of immune sera (unpublished data). Such a functional *in vitro* potential capacity of anti PfMSP5 Ab could parallel /complement PfMSP1p19 IgG and warrants to be further explored.

In two different transmission settings, anti-PfMSP5 IgG was correlated with protection from clinical episodes of malaria. This was particularly apparent in the youngest, most vulnerable age groups of each cohort experiencing the overwhelming majority of episodes. However, it was also evident in the intermediate 15–29 y group in the Ndiop-2000 study (data not shown), with a high incidence of clinical cases probably because transmission was particularly efficient that year. The older groups as expected generally had too few cases to permit reliable comparisons.

This is the first epidemiological study in which the PfMSP5 antibody response has been investigated in depth in two settings studied with the same active case detection system using a relatively long-term intensive follow-up protocol. The results provide significant support for the interest of PfMSP5 to be included in a multitarget *P. falciparum* vaccine candidate, one of very few with so little polymorphism, potentially offering the prospect of sustained protection against this accomplished antigenic chameleon.
